# Drug-Repositioning Approach for the Discovery of Anti-Influenza Virus Activity of Japanese Herbal (Kampo) Medicines* In Vitro*: Potent High Activity of Daio-Kanzo-To

**DOI:** 10.1155/2018/6058181

**Published:** 2018-11-21

**Authors:** Ken Watanabe

**Affiliations:** Graduate School of Biomedical Sciences, Nagasaki University, 1-12-4 Sakamoto, Nagasaki, 852-8523, Japan

## Abstract

Influenza virus infections are a serious public health concern throughout the world. Emergence of viral resistance to the currently approved anti-influenza drugs warrants the development of new antiviral agents. Japanese herbal medicines called Kampo are very commonly used as prescription medication in Japan, and Mao-to is known to be effective against influenza that is caused by oseltamivir-resistant viruses. However, influenza-related death occurs mainly among the elderly, and for patients with hypertension and diabetes, Mao-to may cause these diseases to worsen. Therefore, the exploration of more potent and safe Kampo medicines may be a good strategy for developing new influenza medicines. Here cell-based screening of anti-influenza virus activity for 42 approved Kampo medicines was performed using the drug-repositioning approach. As a result, four Kampo medicines were selected as potent anti-influenza agents against the A/WSN/33 strain. It was found that Daio-kanzo-to [50% inhibitory concentration (IC_50_) = 10.5 *μ*g/mL; 50% cytotoxic concentration (CC_50_) = 71.6 *μ*g/mL; selective index = 6.8] is more effective than Mao-to. Daio-kanzo-to and its constituent Japanese Pharmacopoeia (JP) Rhubarb were also effective against H3N2 and H1N1 subtypes of influenza viruses, including oseltamivir-insensitive-2009 pandemic clinical isolates. These data suggest the potential application of Daio-kanzo-to for influenza treatment.

## 1. Introduction

Influenza is caused by infection with influenza virus. The influenza virus binds to sialic acids on the cell surface and is incorporated into the cell via endocytosis. The H+ influx from the endosome into the virion is mediated by the M2 protein [1]. As a result, the viral ribonucleoprotein complex releases into the cytoplasm, then virus transcription and replication occurs. The newly synthesized viral proteins assemble with the newly synthesized viral RNA. Virus assembly occurs on the plasma membrane, and the virus particles are released from the cell by viral neuraminidase (NA) [2].

At present, two classes of anti-influenza viral medicines have been approved: M2 inhibitors (amantadine and rimantadine) and NA inhibitors (oseltamivir and zanamivir) [3]. However, influenza viruses have mutated and acquired resistance to these inhibitors, thereby decreasing their efficacy; for example, most of the isolates that were identified during the 2005/2006 season in the United States [4] and Japan [5] were found to be amantadine resistant. An oseltamivir-resistant, seasonal H1N1 virus spread rapidly worldwide during the 2007/2008 influenza season. Despite the currently circulating 2009 H1N1 pandemic viruses still being NA inhibitor-sensitive, the possibility of them rapidly acquiring resistance is very high. A recent study shows that the number of global annual influenza-associated respiratory deaths is approximately 290,000–650,000 [6]. Therefore, the development of novel anti-influenza agents is urgently required.

Natural products, including medicinal plants and their extracts, are attractive targets for novel antiviral drugs owing to their wide variety of chemical constituents. Current progress on novel antiviral activity from traditional medicinal plants, such as* Aspalathus linearis* (rooibos),* Valeriana fauriei* Briquet,* Alpinia galanga* (galangal), and* Alchemilla mollis* (lady's mantle), has been reported. An extract from the leaves of rooibos can be used to treat insomnia and nervous breakdown [7]. A recent study showed that rooibos extract has antihuman immunodeficiency virus (HIV) [8, 9] and anti-influenza activities [10]. It has been reported that crude drugs Japanese Valerian and galangal were traditionally used as stomach medicine and sedatives; their constituents valtrate and 1'-acetoxychavicol have anti-influenza virus activity with nuclear export inhibition of the ribonucleoprotein complex [11]. In addition, it was found that lady's mantle (which is used for hemostasis and alleviating nausea and vomiting) has anti-influenza activity with virucidal effects [12]. These observations show the potential of random screening medicinal plants for the discovery of novel antiviral agents, regardless of the traditional use of the medicinal plants.

To target medicinal plants for random screening, the author focused on Kampo medicines—traditional Japanese herbal medicines that comprise hot water extracts of several mixed herbs—because Kampo medicines have been used since ancient times and are commercially available in pharmacies as prescription drugs. In addition, unlike other forms of folk medicine, the formula and quality of crude drug components of Kampo medicines are well defined by the Japanese Pharmacopoeia (JP) [13]. At present, 148 formulae of Kampo medicines, used for the treatment of both communicable and noncommunicable diseases, are used as prescription medicines [14]. No attempts have been made to screen anti-influenza virus activity of these Kampo medicines.

In this study, the anti-influenza virus activity of 42 representative frequently used Kampo medicines was screened using the drug-repositioning approach. Daio-kanzo-to, which is currently used in the treatment of constipation, abnormal intestinal fermentation, dermatitis, and hemorrhoids, was found to possess anti-influenza virus activity against various strains, including H3N2 and 2009 H1N1 pandemic strains with 50% inhibitory concentration (IC_50_) values of 8.0–20.6 *μ*g/mL. Daio-kanzo-to is now used for over-the-counter (OTC) as well as prescription drugs. This is the first report that Daio-kanzo-to, which is not used for communicable diseases, has anti-influenza virus activity.

## 2. Materials and Methods

### 2.1. Preparation of Kampo Medicines and Crude Drugs

An herbal medicine library (INM deposited WAKANYAKU library, Institute of Natural Medicine, University of Toyama) containing extracts based on Kampo formulae and crude drugs was used for the screening. The library includes 42 extracts based on Kampo formulae that were chosen based on scientific importance and annual sales in Japan. All crude drugs used for the preparation of the library were purchased from Tochimoto Tenkaido (Osaka, Japan). Thirty grams of each crude drug was extracted with 300 mL of purified water by gently boiling for 60 min. For extracts based on Kampo formulae, a mixture of crude drugs (double the amount of daily dose) was extracted in 500 mL of purified water by boiling for 17 min, followed by heating for 13 min. The composition of each Kampo formula is available on the database website [15]. The decoction was filtered and freeze-dried to yield a dry extract powder. Each extract was dissolved in ultra-pure water at a concentration of 10 mg/mL and stored at −30°C before use. The voucher specimens of these crude drugs were deposited at the Museum of Materia Medica, Institute of Natural Medicine (TMPW), University of Toyama, Japan. To test anti-influenza virus activity of hit Kampo medicines obtained by the screening, spray-dried granules of the Kampo medicines Mao-to (EK-27, TJ-27 and N27), Kakkon-to (TJ-1), Bofu-tsusho-san (TJ-62), Daio-kanzo-to (TJ-84), and Dai-kenchu-to (TJ-100) were obtained from either Tsumura & Co. (Tokyo, Japan), Kotaro Pharmaceutical Co., Ltd (Osaka, Japan), or Kracie Holdings, Ltd. (Tokyo, Japan). Spray-dried granules of crude drugs (class 2 OTC drug) extracted from JP Glycyrrhiza and JP Rhubarb were obtained from Kracie and Zaiseidoyakuhin Co., Ltd. (Wakayama, Japan), respectively. Detailed information for composition of these Kampo and crude drugs were available in Supplementary Materials (available here).

### 2.2. Chemicals, Cells, and Viruses

Oseltamivir phosphate (F. Hoffmann-La Roche, Basel, Switzerland) was dissolved in phosphate-buffered saline (PBS) at a concentration of 10 mM. Amantadine hydrochloride (Sigma Aldrich, Tokyo, Japan) was dissolved in ultra-pure water at 300 mM. Sennoside A and Sennoside B were obtained from Wako Pure Chemical Industries, Ltd. (Tokyo, Japan) and dissolved in dimethyl sulfoxide (DMSO) at a concentration of 30 mM. All of the compounds were maintained at −30°C until use. Before performing the experiments, the compounds were diluted with Eagle's minimum essential medium (MEM) supplemented with 1% 100 × vitamin solution (MEM vitamin). Madin-Darby canine kidney (MDCK) cells, kindly donated by Dr. Kyosuke Nagata (Tsukuba University, Japan), were grown in MEM supplemented with 5% fetal bovine serum (FBS). These cells were maintained at 37°C in an atmosphere of 5% CO_2_. Influenza viruses, except for A/California/7/2009 (H1N1), were prepared as described previously [16] and stored at −80°C. Allantoic fluid from embryonated eggs of A/California/7/2009 (H1N1) was obtained from Dr. Hiroshi Kido (Tokushima University) and stored at −80°C.

### 2.3. Crystal Violet Assays for Screening and Antiviral Activity Evaluations

The anti-influenza virus activities of samples were evaluated as described previously with some modifications [16]. Briefly, to evaluate the anti-influenza virus activities, MDCK cells seeded into 96-well plates (3.0 × 10^4^ cells/well) were washed with MEM vitamin, the medium was removed, and 100 *μ*L of the serially diluted compound was then added. Cells were subsequently added with 100 *μ*L of virus solution in MEM vitamin equivalent to 100 median tissue culture infectious dose (TCID_50_). The culture plates were incubated at 37°C for 48 h. After incubation, cells were fixed with 70% EtOH and stained with 0.5% crystal violet (CV). After washing and air drying, absorbance was measured at 560 nm using an Infinite M200 pro plate reader (Tecan Japan Co. Ltd, Kanagawa, Japan). The percentage of relative cell density was calculated by comparing the optical density of the treated wells to those of the untreated controls. For screening experiments, Kampo medicines and plant extracts that yielded >30% relative cell density were considered to have antiviral activity. For determination of IC_50_ values of samples, relative cell density was calculated from the dose-response curve by linear regression analysis. For determination of 50% cytotoxic concentration (CC_50_) values of samples, samples were prepared as above except for virus in the MEM vitamin solution was omitted. The selectivity index was calculated using the formula: CC_50_/IC_50_.

### 2.4. Virus Titration

Virus titer in the culture supernatant of cells infected with the virus in the presence of each Kampo medicine was determined using TCID_50_ assay as described [17]. Briefly, the culture supernatant was transferred to 96-well microtiter plates and serially diluted 10-fold in MEM vitamin. A hundred microliters of the dilutions was added to MDCK cells seeded in 96-well tissue culture plates. After incubation for 72 h, cell density was determined by CV staining and TCID_50_ per virus dilution calculated using Reed and Muench Method.

### 2.5. Statistical Analysis

The results in tables represented as mean ± standard deviation were calculated from three independent experiments performed in duplicate.

## 3. Results

### 3.1. Cell-Based Screening of Anti-Influenza Virus Activity of Kampo Medicines

**Figure 1:**
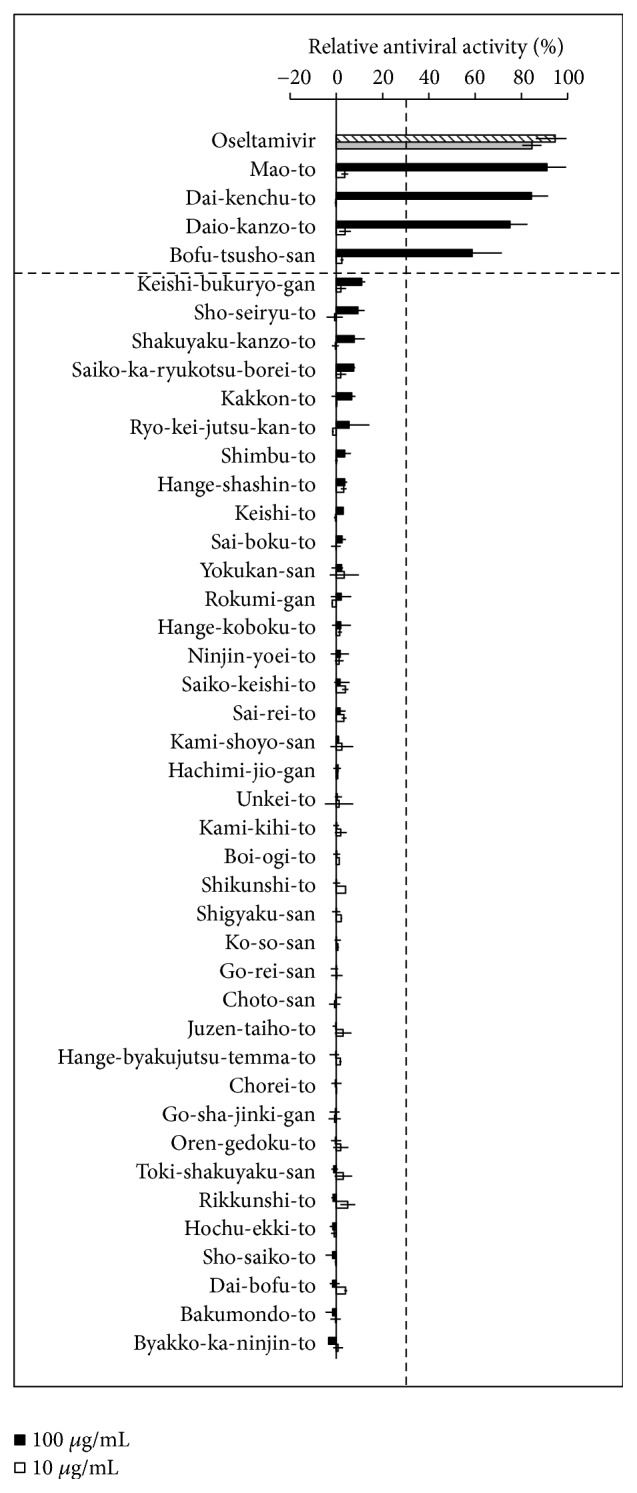
Cell-based screening of anti-influenza virus activity from Kampo medicines. Kampo medicines were tested to determine their anti-influenza virus activities using the herbal medicine library. Diluted Kampo medicines (closed boxes; 100 *μ*g/mL, open boxes; 10 *μ*g/mL) or oseltamivir (striped box; 41 *μ*g /mL, gray box; 4.1 *μ*g /mL) were added to MDCK cells in the presence of influenza virus A/WSN/33 and incubated for 48 h. The cells were fixed and stained with CV. Kampo medicines with antiviral activities > 30% were considered to be positive results. Average values and standard deviation from two independent experiments are shown.

A series of Kampo medicines were subjected to cell-based screening using CV assays, which reflected the virus infection-induced cytopathic effects (CPE) in cells (Figure 1). In this assay, when cells were infected with the virus, the cells detached from the bottom of the dish because of the appearance of CPE. Addition of the sample with antiviral activity suppressed CPE. This assay was performed using the herbal medicine library with the standard laboratory influenza virus strain A/WSN/33 (H1N1 subtype) infection. Of the 42 Kampo medicines tested, four showed significant antiviral activity: Mao-to, Bofu-tsusho-san, Daio-kanzo-to, and Dai-kenchu-to. The positive control oseltamivir showed antiviral activity at 41 and 4.1 *μ*g/mL.

### 3.2. Daio-Kanzo-To Mao-To, Bofu-Tsusho-San, and Kakkon-To Suppress Influenza Virus Propagation

**Figure 2:**
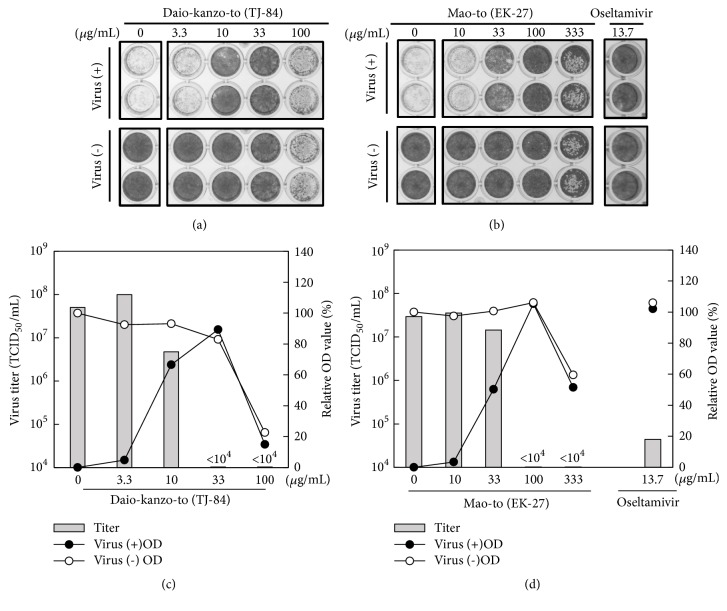
Anti-influenza virus activity of commercially available Daio-kanzo-to and Mao-to. (a) and (b) show representative data from three independent experiments. ((a), (b)) Cytotoxicity and anti-influenza virus activity of Daio-kanzo-to and Mao-to. MDCK cells in a 96-well culture plate, treated with serial dilutions of Daio-kanzo-to (TJ-84) or Mao-to (EK-27), were incubated at 37°C for 48 h with or without A/WSN/33 virus. Oseltamivir (13.6 *μ*g/mL) was used as a control. The cells were stained by CV. ((c), (d)) Culture supernatants and CV stained results in (a) and (b) were used for determination of virus titer and relative OD values, respectively. For determination of virus titer, culture supernatants were collected before fixation and the virus titers (gray rectangles) were determined by the TCID_50_ method. Relative OD values in the presence (closed circles) or absence (open circles) of virus was calculated with reference to uninfected untreated control. Average values from duplicate measurements are shown.

The anti-influenza virus activities of four Kampo medicines obtained by the screening with the herbal medicine library were confirmed using commercially available Kampo medicines and IC_50_ values were determined using serially diluted samples (Table 1). Surprisingly, the IC_50_ value of Daio-kanzo-to (10.5 *μ*g/mL) was stronger than that of Mao-to (IC_50_ value: 45.6–84.1 *μ*g/mL), which is well known for its strong anti-influenza virus activity. Bofu-tsusho-san showed relatively weak anti-influenza virus activity (IC_50_ value: 170 *μ*g/mL). The anti-influenza virus activity of Kakkon-to was also tested because Kakkon-to is a well-known Kampo medicine used for the treatment of flu-like symptoms [18]. Kakkon-to suppressed the appearance of CPE in influenza virus in the CV assay. The IC_50_ value of Kakkon-to (175.7 *μ*g/mL) was higher than the screening concentration (Figure 1, 100 *μ*g/mL), which is why Kakkon-to was not active in the first screening. Next, antiviral activity of the most potent Kampo medicine tested, Daio-kanzo-to, was confirmed by measuring virus titer (Figure 2). Mao-to (EK-27) and oseltamivir were used as controls (Figures 2(b) and 2(d)). Virus titer in the culture supernatant was markedly reduced (<1 × 10^4^ TCID_50_/mL, Figure 2(c)) by 33.3 *μ*g/mL of Daio-kanzo-to treatment where relative OD value of CV assay is good, suggesting that Daio-kanzo-to efficiently suppresses virus propagation.

### 3.3. Determination of Active Constituents in Kampo Medicines

**Figure 3:**
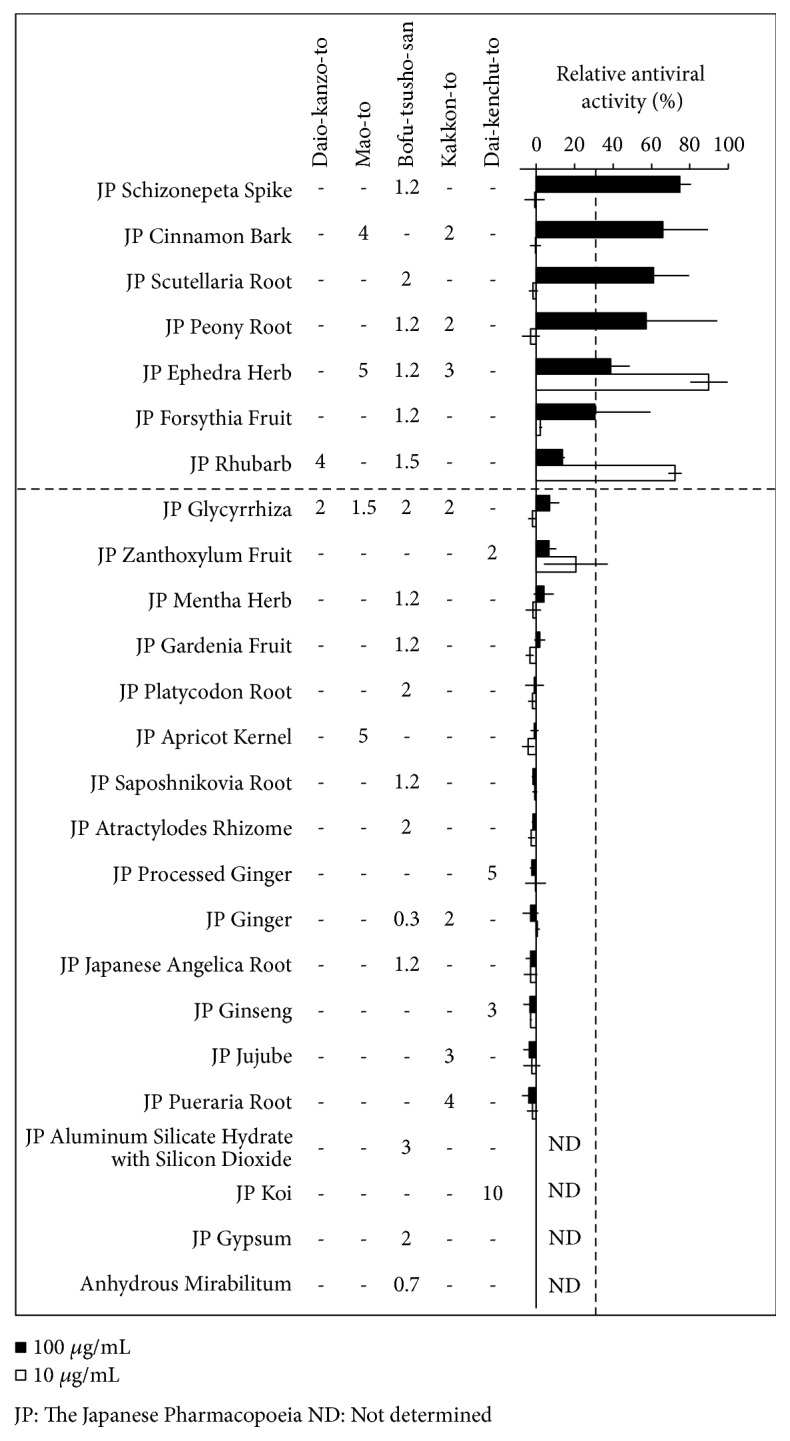
The quantity of crude drugs for Kampo medicines and their anti-influenza virus activity. The quantity of crude drugs (g) for daily dose in Daio-kanzo-to (TJ-84), Mao-to (EK-27, TJ-27, and N27), Bofu-tsusho-san (TJ-62), Kakkon-to (TJ-1), and Dai-kenchu-to (TJ-100) are shown. Crude drugs in the herbal medicine library were used in the screening. Crude drugs were added to MDCK cells in the presence of influenza virus A/WSN/33 and incubated for 48 h. The cells were fixed and stained with CV. Relative anti-influenza virus activities of crude drugs at 100 and 10 *μ*g/mL are shown as closed and open boxes, respectively. Crude drugs with antiviral activities >30% were considered positive results. Average values and standard deviation from two independent experiments are shown.

Next, crude drugs that are responsible for the anti-influenza virus activity of Kampo medicines were screened using the herbal medicine library (Figure 3). Daio-kanzo-to is composed of two crude drugs: JP Rhubarb and JP Glycyrrhiza. Only JP Rhubarb showed antiviral activity. Among the four crude drugs present in Mao-to, JP Cinnamon Bark and JP Ephedra Herb had anti-influenza virus activity. Similarly, 6 out of 17 crude drugs from Bofu-tsusho-san showed antiviral activity. JP Rhubarb, which is a constituent of Daio-kanzo-to and Bofu-tsusho-san, showed good antiviral activity at 10 *μ*g/mL as did JP Ephedra Herb. Dai-kenchu-to contains four crude drugs. Three of these showed no antiviral activity at the concentration tested. Dai-kenchu-to also contains the additive JP Koi (hydrolyzed starch from cereals), which is unlikely to have antiviral activity. Overall, JP Ephedra Herb in Mao-to and JP Rhubarb in Daio-kanzo-to showed strong anti-influenza virus activity at 10 *μ*g/mL. Daio-kanzo-to was selected for further analyses, since its IC_50_ value is greater than Mao-to's (Table 1) and the anti-influenza virus activity of Mao-to is well known and characterized [19–21].

### 3.4. Antiviral Effect of Daio-Kanzo-To and Its Constituents on Various Influenza Viruses

The antiviral effect of Daio-kanzo-to and its constituents, JP Rhubarb and JP Glycyrrhiza, on various influenza virus strains was tested using commercially available crude drugs (Table 2). The IC_50_ values of oseltamivir are varied among viruses tested, as reported previously [17]. Amantadine, the 1^st^ generation anti-influenza drug, was only effective against the H3N2 subtype, with an IC_50_ value of 0.1 *μ*g/mL, whereas it was ineffective against H1N1 subtypes, including clinical isolates (IC_50_ >188 *μ*g/mL). In contrast, Daio-kanzo-to and JP Rhubarb extract alone were found to have a broad antiviral spectrum with similar IC_50_ values against all viral strains, including H1N1 laboratory strains (A/WSN/33 and A/Puerto Rico/8/34), a H3N2 laboratory strain (A/Aichi/2/68), and H1N1 clinical isolates (A/California/7/2009 and A/Virginia/ATCC2/2009). It should be noted that the A/California/7/2009 strain was selected as an antigen for influenza vaccine production in Japan for 7 years (2010-2017), suggesting the potential utility of Daio-kanzo-to. In addition, Daio-kanzo-to and JP Rhubarb extract showed good IC_50_ values in A/Virginia/ATCC2/2009, which has low sensitivity to oseltamivir (IC_50_ >41 *μ*g/mL). Because Sennoside A/B is the most important constituent of Daio-kanzo-to and its quantity in the Rhubarb used for Kampo medicine is defined by the Japanese Pharmacopoeia, the anti-influenza virus activities of Sennoside A/B were tested. Sennoside A/B did not show anti-influenza activity under our experimental conditions (IC_50_ >173 *μ*g/mL), although anti-HIV-1 activity has been reported [22]. JP Glycyrrhiza extract showed no antiviral activity (IC_50_ >100 *μ*g/mL) for all viral strains tested. These results suggest that the anti-influenza virus activity of Daio-kanzo-to is derived from its Rhubarb constituent.

## 4. Discussion

In this paper, the author screened the anti-influenza virus activities of Kampo prescription medicines and found that four Kampo medicines: Mao-to, Daio-kanzo-to, Bofu-tsusho-san and Dai-kenchu-to have antiviral activity (Figure 1). Mao-to has historically been used to alleviate flu-like symptoms, including rigors, fever, headache, and lower-back pain. Daio-kanzo-to and Bofu-tsusho-san are currently used to treat constipation. In traditional use of Kampo medicine, an old piece of literature, Shokan-zatsubyo-ron, published in 220 AD, suggested that Mao-to and Kakkon-to are applicable for the treatment of infectious diseases such as respiratory tract infection and influenza [23]. The antiviral activity of Dai-kenchu-to was not confirmed when the spray-dried commercially available medicine was used (IC_50_ >1000 *μ*g/mL, Table 1). This may be due to the antiviral activity of this aromatic compound being easily lost under the heat treatment stage of the spray-drying process.

Utilization of Kampo medicines for the treatment of flu is a good strategy to avoid the emergence of drug-resistant viruses. Several JP crude drugs that have anti-influenza virus activity were commonly contained in the four Kampo medicines that were identified in the present study as having anti-influenza virus activities. Mao-to, Kakkon-to and Bofu-tsusho-san all contain Ephedra Herb. It has been reported that Ephedra Herb acts in the early stage of the influenza virus life cycle [24]. Cinnamon Bark is present in Mao-to and Kakkon-to. A component of Cinnamon Bark,* trans-*cinnamaldehyde, has been shown to have anti-influenza virus activity in the mid-stage of the virus life cycle [25]. Daio-kanzo-to and Bofu-tsusho-san contain Rhubarb. It is has been reported that Rhubarb contains anthraquinone compounds, which have been reported to have antiviral activity against various viruses with virucidal effects [26]. Peony Root is reported to have anti-influenza virus activity at the point of virus entry [27] and is present in Bofu-tsusho-san and Kakkon-to.

There are some discrepancies regarding anti-influenza virus activity between Kampo formula and ingredient crude drugs. Dai-kenchu-to showed antivirus activity (Figure 1) although any of the ingredient crude drugs had no activity (Figure 3). This is may be due to the synergistic effect of combination of crude drugs. Sho-seiryu-to, Saiko-ka-ryukotsu-borei-to, and Kakkon-to did not show any antivirus activity (Figure 1), although they contain Ephedra Herb or Rhubarb. One possible explanation is that these Kampo medicines contain large amount of crude drugs that do not have anti-influenza virus activity; hence, relative amount of active ingredient in the extract could be low. The relative amount of Ephedra Herb in Mao-to is 32.3%, whereas that in Sho-seiryu-to and Kakkon-to is 11% and 15%, respectively. Similarly, the relative amount of Rhubarb in Daio-kanzo-to is 66.6%, whereas that in Saiko-ka-ryukotsu-borei-to is 3.9%. Another explanation is that the extraction condition of a crude drug alone and mixture of crude drugs as Kampo formulae might be different. For example, it has been reported that Oyster Shell in Saiko-ka-ryukotu-borei-to affects the extraction efficacy of some ingredients such as baicalin and saikosaponin due to relatively higher pH value and calcium contents [28].

It is generally accepted that the combined use of antiviral components with different mechanisms of action can avoid the emergence of drug-resistant viruses and increase drug efficacy synergistically. Since these Kampo medicines contain several crude drugs with different mechanisms of action of antiviral activity, it is expected that they could help reduce the emergence of drug-resistant viruses. Indeed, in Japan, patients sometimes take Kampo medicines in combination with approved antiviral drugs, for example, Mao-to and oseltamivir [21]. Similarly, Daio-kanzo-to can be used with neuraminidase inhibitors to avoid the emergence of drug-resistant viruses.

Recent reports show that Kampo medicine is effective for the treatment of flu-like symptoms. Mao-to is effective for influenza infection* in vitro* [20] and* in vivo *[19] and has been widely used for the treatment of flu-like illness, based on prescription in Japan. In addition, it has been reported that Mao-to is as effective as oseltamivir for the treatment of type A influenza infection in adults [29] and children [21]. Kakkon-to has been shown to have anti-influenza virus activity* in vitro* via the PI3K/AKT pathway [30] and* in vivo* [18]. These results clearly show that the potential of Kampo medicines for the treatment of influenza. However, according to the latest JP (17^th^ edition) [13], Ephedra Herb for Mao-to and Kakkon-to should include a significant amount (> 0.7%) of ephedrine alkaloids. The use of these Kampo medicines for older patients should be cautious, since ephedrine alkaloids are known to exacerbate hypertension and diabetes, as cautioned in the package insert of Mao-to. A recent study suggests that influenza infection in patients aged 75 years or older carries a high risk of mortality in Japan [6]. Therefore, Kampo medicines that are applicable for older patients, who have hypertension or diabetes, are highly needed. Daio-kanzo-to is used as a prescription drug and is also widely available as an OTC Kampo drug in Japan. Our results show that Daio-kanzo-to is more potent than Mao-to (Table 1) and is effective against currently circulating 2009 pandemic viruses, including an oseltamivir-insensitive strain (A/Virginia/ATCC2/2009 strain, IC_50_ of oseltamivir >41 *μ*g/mL, Table 2). These results suggest that Daio-kanzo-to could be effective for influenza treatment.

In considering the application of Daio-kanzo-to as an anti-influenza drug, the strong cathartic effect derived from components of Rhubarb sennosides could be a problem. Several lines of the report show that number of constituents in Kampo medicines can be controlled by processing methods. Old Chinese literature suggests that Rhubarb was originally used as stomach medicine and the processing method of Rhubarb was heat-drying [31]. Yoneda* et al.* [32] and Yoshida* et al.* [33] have reported that the level of sennosides in Rhubarb tended to decrease with heat-drying, whereas the level of anthraquinones, the decomposition product of sennosides, increased. The virucidal effects of anthraquinones have been reported [26, 34]. Our results suggest that Sennnoside A and B does not have anti-influenza virus activity (Table 2); therefore Rhubarb, which contains a small amount of sennosides, could be a good material for production of Daio-kanzo-to. Identification of the active components of Rhubarb is ongoing.

In summary, Daio-kanzo-to and JP Rhubarb were found to possess anti-influenza virus activities. Further studies are necessary to test activity against various influenza viruses such as type B strains and to elucidate their effects* in vivo*.

## Figures and Tables

**Table 1:** Anti-influenza virus activity of commercially available Kampo medicines.

	IC_50_ (*μ*g/mL)^a^	CC_50_ (*μ*g/mL)^b^	SI^c^	Remarks
Daio-kanzo-to (TJ-84)	10.5±5.5	71.6±9.1	6.8	Tsumura & Co.
Mao-to (EK-27)	45.6±11.3	>1000	>21.9	Kracie Holdings, Ltd.
Mao-to (TJ-27)	53.3±0.7	264.2±15.6	5.0	Tsumura & Co.
Mao-to (N27)	84.1±24.1	>1000	>11.9	Kotaro pharmaceutical Co., Ltd.
Bofu-tsusho-san (TJ-62)	170.0±1.5	>333.3	>2.0	Tsumura & Co.
Kakkon-to (TJ-1)	175.7±16.9	>333.3	>1.9	Tsumura & Co.
Dai-kenchu-to (TJ-100)	>1000	>1000	N/A^d^	Tsumura & Co.
Oseltamivir	2.1±0.4	>137	>65.2	

^a^50% inhibitory concentration against A/WSN/33 virus.

^b^50% cytotoxic concentration for MDCK cells.

^c^The ratio of CC_50_ to IC_50_.

^d^Not applicable.

**Table 2:** Antiviral effect of Daio-kanzo-to and its constituents on various influenza viruses.

		IC_50_ (*μ*g/mL)^a^						
Strain	Subtype	Daio-kanzo-to (TJ-84)	JP^b^ Rhubarb extract^c^	JP Glycyrrhiza extract^d^	Sennoside A	Sennoside B	Oseltamivir	Amantadine
A/WSN/33	H1N1	10.5±5.5	10.4±6.2	>100	>173	>173	2.1±0.4	80.9±32.6
A/Puerto Rico/8/34	H1N1	8.0±0.2	21.6±1.5	>100	>173	>173	2.0±0.2	>188
A/Aichi/2/68	H3N2	15.9±7.3	20.9±1.2	>100	>173	>173	0.4±0.1	0.1±0.0
A/California/7/2009	H1N1	16.7±3.6	20.0±4.4	>100	>173	>173	0.4±0.0	>188
A/Virginia/ATCC2/2009	H1N1	20.6±4.2	19.1±2.1	>100	>173	>173	>41.0	>188

^a^50% inhibitory concentration.

^b^The Japanese Pharmacopoeia.

^c^Commercially available Rhubarb crude drug (Zaiseidoyakuhin Co., Ltd.)

^d^Commercially available Glycyrrhiza crude drug (Kracie Holdings, Ltd.).

## Data Availability

The data used to support the findings of this study are included within the article.

## Supplementary Materials

Supplementary Materials Composition of Kampo medicines and crude drugs used in this study are described in Supplementary Materials and Methods.
