# Supplementary material for: Drug-Repositioning Approach for the Discovery of Anti-Influenza Virus Activity of Japanese Herbal (Kampo) Medicines In Vitro: Potent High Activity of Daio-Kanzo-To
Source: Evid Based Complement Alternat Med. 2018 Nov 21;2018:6058181. doi: 10.1155/2018/6058181 (PMC6280228; doi:10.1155/2018/6058181)
Supplement: Supplementary Materials — Composition of Kampo medicines and crude drugs used in this study are described in Supplementary Materials and Methods. [file 6058181.f1.pdf]

Supplemental Material

Drug-Repositioning Approach for the Discovery of Anti-influenza Virus Activity of Japanese Herbal (Kampo) Medicines *in vitro*: Potent High Activity of Daio-kanzo-to

Author: Ken Watanabe

Supplementary Materials and Methods

1. Description of Kampo medicines used for the screening (Figure 1)

Weigh 2 times the daily dose of each ingredient (crude drug) of a Kampo formula. Put all the ingredients into a decoction equipment (UIS-1900, Uchida Wakanyaku, Co., Ltd.), and then add 500 mL of water for extraction. Bring the water to boil over high heat for 17 minutes and then continuously simmer it on low heat for 13 minutes without cover. Filter the solution with a tea strainer. Leave the solution to reach the room temperature, freeze-dry it and reconstituted at 10 mg/mL with water. Description of Kampo medicines used for the screening were as follows.

Daily dose of Mao-to: 6 g of Apricot Kernel, 6 g of Ephedra Herb, 3 g of Cinnamon Bark and 1.5 g of Glycyrrhiza.

Daily dose of Dai-kenchu-to: 3 g of Ginseng, 2.5 g of Zanthoxysum Fruit and 1.5 g of Processed Ginger.

Daily dose of Daio-kanzo-to: 2g of Glycyrrhiza and 1 g of Rhubarb.

Daily dose of Bofu-tsusho-san: 5 g of Aluminum Silicate Hydrate with Silicon Dioxide, 3 g of Gypsum, 2.5 g of Scutellaria Root, 2 g of Gardenia Fruit, 2 g of Atractylodes Rhizome, 1.2 g of Sodium Sulfate, 1.2 g of Peony Root, 1.2 g of Cnidium Rhizome, 1.2 g of Japanese Angelica Root, 1.2 g of Mentha Herb, 1.2 g of Ephedra Herb, 1.2 g of Forsythia Fruit, 1.2g of Saposhnikovia Root and Rhizome, 1 g of Ginger and 2 g of Rhubarb.

Daily dose of Keishi-bukuryo-gan: 4 g of Cinnamon Bark, 4 g of Peony Root, 4 g of Peach Kernel, 4 g of Poria Sclerotium and 4 g of Montan Bark.

Daily dose of Sho-seiryu-to: 6g of Pinellia Tuber, 3g of Glycyrrhiza, 3 g of Cinnamon Bark, 3 g of Schisandra Fruit, 3 g of Asiarum Root, 3 g of Peony Root, 3 g of Ephedra Herb and 1.5 g of Processed Ginger.

Daily dose of Shakuyaku-kanzo-to: 3 g of Glycyrrhiza, and 3 g of Peony Root.

Daily dose of Saiko-ka-ryukotsu-borei-to: 7 g of Bupleurum Root, 5 g of Pinellia Tuber, 3 g of Scutellaria Root, 3 g of Cinnamon Bark, 3 g of Jujube, 3 g of Ginseng, 3 g of Poria Sclerotium, 3 g of Oyster Shell, 3 g of Longgu, 1 g of Ginger and 1 g of Rhubarb.

Daily dose of Kakkon-to: 8 g of Pueraria Root, 4 g of Jujube, 4 g of Ephedra Herb, 3 g of Cinnamon Bark, 3 g of Peony Root 2 g of Glycyrrhiza and 1 g of Ginger.

Daily dose of Ryo-kei-jutsu-kan-to: 8 g of Poria Sclerotium, 5 g of Cinnamon Bark, 4 g of Atractylodes Rhizome and 3 g of Glycyrrhiza.

Daily dose of Shimbu-to: 6 g of Poria Sclerotium, 5 g of Peony Root, 4 g of Atractylodes Rhizome, 1 g of Ginger and 1 g of Processed Aconite Root.

Daily dose of Hange-shashin-to: 7 g of Pinellia Tuber, 3 g of Scutellaria Root, 3 g of Jujube, 3 g of Ginseng, 2.5 g of Glycyrrhiza, 1.5 g of Processed Ginger and 1 g of Coptis Rhizome.

Daily dose of Keishi-to: 6 g of Cinnamon Bark, 6 g of Peony Root, 6 g of Jujube, 2.5 g of Glycyrrhiza and 1 g of Ginger.

Daily dose of Sai-boku-to: 7 g of Bupleurum Root, 5 g of Pinellia Tuber, 5 g of Poria Sclerotium, 3 g of Scutellaria Root, 3 g of Magnolia Bark, 3 g of Jujube, 3 g of Ginseng, 2 g of Glycyrrhiza, 2 g of Perilla Herb and 1 g of Ginger.

Daily dose of Yokukan-san: 4 g of Uncaria Hook, 4 g of Japanese Angelica Root, 4 g of Atractylodes Rhizome, 4 g of Poria Sclerotium, 3 g of Bupleurum Root, 3 g of Cnidium Rhizome and 1.5 g of Glycyrrhiza.

Daily dose of Rokumi-gan: 8 g of Rehmannia Root, 4 g of Cornus Fruit, 4 g of Dioscorea Rhizome, 3 g of Alisma Rhizome, 3 g of Poria Sclerotium and 3 g of Moutan Bark.

Daily dose of Hange-kobuku-to: 8 g of Pinellia Tuber, 5 g of Poria Sclerotium, 3 g of Magnolia Bark, 2 g of Perilla Herb, and 1 g of Ginger.

Daily dose of Ninjin-yoei-to: 4 g of Rehmannia Root, 4 g of Japanese Angelica Root, 4 g of Atractylodes Rhizome, 4 g of Poria Sclerotium, 3 g of Ginseng, 2.5 g of Cinnamon

Bark, 2 g of Astragalus Root, 2 g of Polygala Root, 2 g of Peony Root, 2 g of Citrus Unshiu Peel, 1 g of Glycyrrhiza and 1 g of Schisandra Fruit.

Daily dose of Saiko-keishi-to: 6 g of Bupleurum Root, 4.5 g of Pinellia Tuber, 2.5 g of Scutellaria Root, 2.5 g of Cinnamon Bark, 2.5 g of Peony Root, 2.5 g of Jujube, 2.5 g of Ginseng, 1.5 g of Glycyrrhiza and 1 g of Ginger.

Daily dose of Sai-rei-to: 5 g of Bupleurum Root, 4 g of Alisma Rhizome, 4 g of Pinellia Tuber, 3 g of Polyporus Sclerotium, 3 g of Atractylodes Rhizome, 3 g of Poria Sclerotium, 2.5 g of Scutellaria Root, 2.5 g of Cinnamon Bark, 2.5 g of Jujube, 2.5 g of Ginseng, 2 g of Glycyrrhiza and 1 g of Ginger.

Daily dose of Daily dose of Kami-shoyo-san: 3 g of Bupleurum Root, 3 g of Peony Root, 3 g of Japanese Angelica Root, 3 g of Atractylodes Rhizome, 3 g of Poria Sclerotium, 2 g of Glycyrrhiza, 2 g of Gardenia Fruit, 2 g of Montan bark 1 g of Ginger and 1 g of Mentha Herb.

Daily dose of Hachimi-jio-gan: 6 g of Rehmannia Root, 3.5 g of Cornus Fruit, 3.5 g of Dioscorea Rhizome, 3 g of Alisma Rhizome, 3 g of Poria Sclerotium, 3 g of Moutan Bark, 1 g of Cinnamon Bark and 1 g of Processed Aconite Root.

Daily dose of Unkei-to: 5 g of Ophiopogon Tuber, 4 g of Pinellia Tuber, 3 g of Japanese Angelica Root, 2 g of Glycyrrhiza, 2 g of Cinnamon Bark, 2 g of Euodia Fruit, 2 g of Peony Root, 2 g of Cnidium Rhizome, 2 g of Ginseng, 2 g of Moutan Bark, 1.5 g of Donkey Glue and 1 g of Ginger.

Daily dose of Kami-kihi-to: 3 g of Bupleurum Root, 3 g of Jujube Seed, 3 g of Ginseng, 3 g of Atractylodes Rhizome, 3 g of Poria Sclerotium, 3 g of Longan Aril, 2 g of Astragalus Root, 2 g of Gardenia Fruit, 2 g of Japanese Angelica Root, 1.5 g of Polygala Root, 1.5 g of Jujube, 1 g of Glycyrrhiza, 1 g of Ginger and 1 g of Saussurea Root.

Daily dose of Boi-ogi-to: 5 g of Astragalus Root, 4 g of Sinomenium Stem and Rhizome, 3 g of Atractylodes Rhizome, 3 g of Jujube, 2 g of Glycyrrhiza and 1 g of Ginger.

Daily dose of Shikunshi-to: Ginseng, 4 g of Atractylodes Rhizome, 4 g of Poria Sclerotium, 2 g of Glycyrrhiza, 2 g of Jujube and 1 g of Ginger.

Daily dose of Shigyaku-san: 5 g of Bupleurum Root , 5 g of Peony Root, 4 g of Immature Orange and 3 g of Glycyrrhiza.

Daily dose of Ko-so-san: 4 g of Cyprus Rhizome, 2 g of Perilla Herb, 2 g of Citrus Unshiu Peel, 1.5 g of Glycyrrhiza and 1 g of Ginger.

Daily dose of Go-rei-san: 6 g of Alisma Rhizome, 4.5 g of Polyporus Sclerotium, 4.5 g of Atractylodes Rhizome, 4.5 g of Poria Sclerotium and 3 g of Cinnamon Bark.

Daily dose of Choto-san: 5 g of Gypsum, 3 g of Chrysanthemum Flower, 3 g of Uncaria Hook, 3 g of Citrus Unshiu Peel, 3 g of Ginseng, 3 g of Pinellia Tuber, 3 g of Ophiopogon Tuber, 3 g of Poria Sclerotium, 3 g of Saposhnikovia Root and Rhizome, 1 g of Glycyrrhiza and 1 g of Ginger.

Daily dose of Juzen-taiho-to: 3 g of Astragalus Root, 3 g of Cinnamon Bark, 3 g of Peony Root, 3 g of Processed Rehmannia Root, 3 g of Cnidium Rhizome, 3 g of Japanese Angelica Root, 3 g of Ginseng, 3 g of Atractylodes Rhizome, 3 g of Poria Sclerotium and 1.5 g of Glycyrrhiza.

Daily dose of Hange-byakujutsu-temma-to: 3 g of Citrus Unshiu Peel, 3 g of Malt, 3 g of Pinellia Tuber, 2 g of Astragalus Root, 2 g of Atractylodes Lancea Rhizome, 2 g of Alisma Rhizome, 2 g of Gastrodia Tuber, 2 g of Ginseng, 2 g of Poria Sclerotium, 1.5 g of Shinkiku, 1.5 g of Atractylodes Rhizome, 1 g of Phellodendron Bark, 1 g of Processed Ginger and 0.5 g of Ginger.

Daily dose of Chorei-to: 4 g of Aluminum Silicate Hydrate with Silicon Dioxide, 4 g of Alisma Rhizome, 4 g of Polyporus Sclerotium, 4 g of Poria Sclerotium and 1.5 g of Donkey Glue.

Daily dose of Go-sya-jinki-gan: 5 g of Rehmannia Root, 3 g of Achyranthes Root, 3 g of Cornus Fruit, 3 g of Dioscorea Rhizome, 3 g of Alisma Rhizome, 3 g of Poria Sclerotium, 3 g of Moutan Bark, 2 g of Plantago Seed, 1 g of Cinnamon Bark and 1 g of Processed Aconite Root.

Daily dose of Oren-gedoku-to: 3 g of Coptis Rhizome, 2 g of Scutellaria Root, 2 g of Phellodendron Bark and 2 g of Gardenia Fruit.

Daily dose of Toki-syakuyaku-san: 6 g of Alisma Rhizome, 5 g of Peony Root, 4 g of Atractylodes Rhizome, 4 g of Poria Sclerotium, 3 g of Cnidium Rhizome and 3 g of Japanese Angelica Root.

Daily dose of Rikkunshi-to: 4 g of Ginseng, 4 g of Pinellia Tuber, 4 g of Atractylodes Rhizome, 4 g of Poria Sclerotium, 2 g of Jujube, 2 g of Citrus Unshiu Peel, 1 g of Glycyrrhiza and 0.5 g of Ginger.

Daily dose of Hochu-ekki-to: 4 g of Astragalus Root, 4 g of Japanese Angelica Root, 4 g of Ginseng, 4 g of Atractylodes Rhizome, 2 g of Bupleurum Root, 2 g of Cimicifuga Rhizome, 2 g of Jujube, 2 g of Citrus Unshiu Peel, 1.5 g of Glycyrrhiza and 1 g of Ginger.

Daily dose of Sho-saiko-to: 8 g of Bupleurum Root, 6 g of Pinellia Tuber, 3 g of Scutellaria Root, 3 g of Jujube, 3 g of Ginseng, 2.5 g of Glycyrrhiza and 1 g of Ginger.

Daily dose of Dai-bofu-to: 3 g of Astragalus Root, 3 g of Peony Root, 3 g of Processed Rehmannia Root, 3 g of Japanese Angelica Root, 3 g of Eucommia Bark, 3 g of Atractylodes Rhizome, 3 g of Saposhnikovia Root and Rhizome, 2 g of Cnidium Rhizome, 1.5 g of Glycyrrhiza, 1.5 g of Notopterygium, 1.5 g of Achyranthes Root, 1.5 g of Jujube, 1.5 g of Ginseng, 1 g of Processed Ginger and 1 g of Processed Aconite Root.

Daily dose of Bakumondo-to: 10 g of Ophiopogon Tuber, 5 g of Brown Rice, 5 g of Pinellia Tuber, 3 g of Jujube, 2 g of Glycyrrhiza and 2 g of Ginseng.

Daily dose of Byakko-ka-ninjin-to: 15 g of Gypsum, 9 g of Brown Rice, 5 g of Anemarrhena Rhizome, 3 g of Ginseng and 2 g of Glycyrrhiza.

2. *Description of commercially available Kampo medicines and crude drugs.*

7.5 g (daily dose) of Tsumura Kakkon-to extract granules (TJ-1) contains 3.75 g of a dried extract of the following mixed crude drugs. JP Pueraria Root 4.0 g, JP Jujube 3.0 g, JP Ephedra Herb 3.0 g, JP Glycyrrhiza 2.0 g, JP Cinnamon Bark 2.0 g, JP Peony Root 2.0

g, and JP Ginger 2.0 g (JP: The Japanese Pharma copoeia).

7.5 g (daily dose) of Tsumura Mao-to extract granules (TJ-27) contains 1.75 g of a dried extract of the following mixed crude drugs. JP Apricot Kernel 5.0 g, JP Ephedra Herb 5.0 g, JP Cinnamon Bark 4.0 g, and JP Glycyrrhiza 1.5 g.

6.0 g (daily dose) of Kotaro Mao-to extract granules (N27) contains 1.9 g of a dried extract of the following mixed crude drugs. JP Apricot Kernel 5.0 g, JP Ephedra Herb 5.0 g, JP Cinnamon Bark 4.0 g, and JP Glycyrrhiza 1.5 g.

6.0 g (daily dose) of Kuracie Mao-to extract granules (EK-27) contains 1.6 g of a dried extract of the following mixed crude drugs. JP Apricot Kernel 5.0 g, JP Ephedra Herb 5.0 g, JP Cinnamon Bark 4.0 g, and JP Glycyrrhiza 1.5 g.

7.5 g (daily dose) of Tsumura Bofu-tsusho-san extract granules (TJ-62) contains 4.5 g of a dried extract of the following mixed crude drugs. JP Alminum Silicate Hydrate with Sili-con Dioxide 3.0 g, JP Scutellaria Root 2.0 g, JP Glycyrrhiza 2.0 g, JP Platycodon Root 2.0 g, JP Gypsum 2.0 g, JP Atractylodes Rhizome 2.0 g, JP Rhubarb 1.5 g, JP Schizonepeta Spike 1.2 g, JP Gardenia Fruit 1.2 g, JP Peony Root 1.2 g, JP Cnidium Rhizome 1.2 g, JP Japanese Angelica Root 1.2 g, JP Mentha Herb 1.2 g, JP Saposhnikovia Root 1.2 g, JP Ephedra Herb 1.2 g, JP Forsythia Fruit 1.2 g, JP Ginger 0.3 g, and Anhydrous Mirabilitum 0.7 g.

7.5 g (daily dose) of Tsumura Daio-kanzo-to extract granules (TJ-84) contains 1.5 g of a dried extract of the following mixed crude drugs. JP Rhubarb 4.0 g, and JP Glycyrrhiza 2.0 g.

15.0 g (daily dose) of Tsumura Dai-kenchu-to extract granules (TJ-100) contains 10.0 g of JP Koi and 1.25 g of a dried extract of the following mixed crude drugs. JP Processed Ginger 5.0 g, JP Ginseng 3.0 g, and JP Zanthoxylum Fruit 2.0 g.

1.5 g (daily dose) of Zaiseidoyakuhin Rhubarb extract granules contains 0.5 g of a dried extract of JP Rhubarb.

1.2g (daily dose) of Kuracie Glycyrrhiza extract granules contains 1.425 g of a dried extract of JP Glycyrrhiza (JP : The Japanese Pharmacopoeia).
